# Multi-Year Assessment of Toxic Genotypes and Microcystin Concentration in Northern Lake Taihu, China

**DOI:** 10.3390/toxins8010023

**Published:** 2016-01-15

**Authors:** Lili Hu, Kun Shan, Lizhou Lin, Wei Shen, Licheng Huang, Nanqin Gan, Lirong Song

**Affiliations:** 1 State Key Laboratory of Freshwater Ecology and Biotechnology, Institute of Hydrobiology, Chinese Academy of Sciences, Wuhan 430072, China; lilihu@escience.cn (L.H.); linlizhou290101@gmail.com (L.L.); faithhlc@163.com (L.H.); gannq@ihb.ac.cn (N.G.); 2 University of Chinese Academy of Sciences, Beijing 100049, China; 3 Institute of Electronic Information Technology, Chongqing Institute of Green and Intelligent Technology, Chinese Academy of Sciences, Chongqing 400714, China; shankun@sina.com; 4 Changzhou Environmental Monitoring Center, Changzhou 213001, China; jsdafengshenwei@gmail.com

**Keywords:** *Microcystis* morphospecies, microcystins, qPCR, nitrogen, Lake Taihu

## Abstract

Lake Taihu is the third-largest freshwater lake in China and has been suffering from cyanobacterial blooms for over two decades. The northern part of the lake, Meiliang Bay, is known to be at high risk of dense and sustained *Microcystis* blooms and toxins. This study aimed to investigate and record the annual and seasonal dynamics of toxic genotype, *Microcystis* morphospecies succession and microcystin variation. It also aimed to find out the underlying driving factors influencing the dynamic changes. Microcystin (MC) and the *Microcystis* genotype were quantified using HPLC and quantitative real-time PCR, respectively. Our study, over three consecutive years, showed that the pattern of morphospecies succession was seasonally distinct and annually consistent. During the same period in 2012, 2013 and 2014, the average MC were, on dry weight basis, 733 μg·g^−1^, 844 μg·g^−1^, 870 μg·g^−1^, respectively. The proportion of toxic *Microcystis* accounted for 41%, 44% and 52%, respectively. Cell bound microcystin was found to correlate with the percentage of toxic *Microcystis.* Based on historical and current data, we conclude that annual bloom toxicity was relatively stable or possibly increased over the last decade.

## 1. Introduction

Toxic cyanobacterial blooms are increasing worldwide and sustaining in large water ecosystems, leading to serious consequences for the supply of drinking water [1]. Among bloom-forming toxic cyanobacteria, *Microcystis* is the most frequently reported genus forming blooms in freshwater systems [2]; it can produce highly stable and potent polypeptides known as microcystins [3,4]. The hepatotoxic microcystins, synthesized by an integrated peptide-polyketide synthetase [5,6], are cyclic peptides and share a common structure {cyclo(-Adda-D-Glu-Mdha-D-Ala-L-X-D-MeAsp-L-Z)}, where X and Z represent variable amino acids [7]. Microcystin-LR (MC-LR) and microcystin-RR (MC-RR) are the most common variants among over 90 microcystin analogues found [8,9]. The former is five times more toxic, as indicated by LD_50_ in mice [10].

*Microcystis* populations in bloom season are usually composed of both toxic and non-toxic strains, with the former capable of producing MC. The development and application of quantitative PCR (qPCR) enable researchers to distinguish between toxic and non-toxic strains, thus promoting studies on the spatial and temporal pattern dynamics of microcystin-producing genotypes, thereby helping to understand the correlation between toxic genotypes and microcystin concentrations [11,12,13]. Most studies have indicated a correlation between the molecular quantification or proportion of toxic genotypes and toxicity (microcystin concentration or/and MC content per *Microcystis* cell) [14,15,16,17]. However, other studies have described a less clear-cut or inconsistent correlation [18,19,20].

Variation in microcystin concentrations per unit biomass can be influenced by the relative abundance of toxic genotypes and/or by the response to physical-chemical factors of individual toxic genotypes. A few studies have specifically focused on microcystin quotas in natural systems. Microcystin quota (µg·g^−1^) was positively correlated with the concentration of soluble reactive phosphorus [21,22], the *Daphnia* biomass [23] and nitrogen [24].

*Microcystis* has several morphotypes whose compositions tend to vary both seasonally and annually in cyanobacterial blooms [25,26,27]. Kurmayer *et al.* [28] and Via-Ordorika *et al.* [29] pointed out that different *Microcystis* morphospecies are likely to possess different toxicity potentials. They suggested that the toxin content of field populations can be assessed by the strain composition according to morphological criteria. Typical morphospecies [25,30] and *Microcystis* colony size [31,32] have also been shown to be correlated with microcystin concentration. It is understandable that the application of the morphotype as an indicator of toxicity for individual lakes should be based on the consistency of successive patterns of morphospecies over a prolonged period.

Lake Taihu, the third-largest freshwater lake in China, with an area of 2338 km^2^, provides drinking water for over ten million residents. The large-scale occurrence of *Microcystis* blooms corresponds with pollutants being discharged into the lake since the 1980s [33]. MC pollution is present across the entire area of Lake Taihu, but the risk is higher in the Northern part than in the center of the lake [34,35,36]. The dynamics of MC concentration are considered to correlate with abiotic factors, such as water temperature [37], nitrate [38] and pH [39], and with biotic factors, such as *Microcystis* biomass [40] and Chl-*a* [41]. However, most studies had been carried out over periods that cover only a bloom cycle or only one year, making it difficult to assess the status or the trend of toxicity over multiple years. The purpose of this study was to investigate the dynamics of toxic *Microcystis*, morphospecies and MCs, attempting to illustrate the associations between toxic *Microcystis*, morphospecies and MCs and the possible driving forces influencing the dominance of toxicity. In so-doing, we hoped to be able to assess the toxicity of blooms over the past decade. A three year long study was conducted in the northern part of Lake Taihu to determine a suite of biotic and abiotic parameters. In addition, experiments were undertaken to detect the toxins of the dominant *Microcystis* morphospecies. It is anticipated that this study will be of importance for mitigation and management of toxic blooms in Lake Taihu.

## 2. Results

### 2.1. Physicochemical Parameters

**Figure 1:**
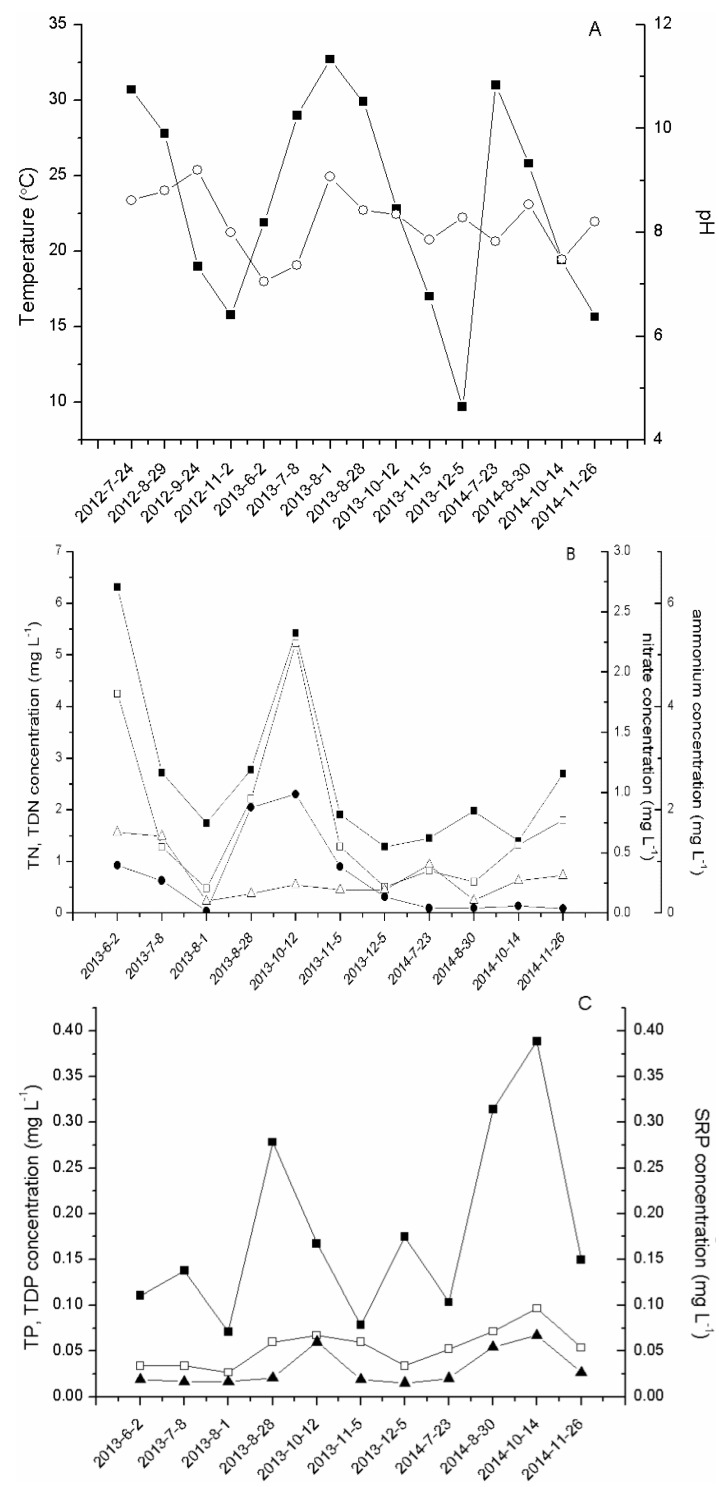
Environmental parameters in Meiliang Bay, N2, of Lake Taihu. (**A**) Temperature (■), pH (о); (**B**) TN (■), TDN (□), NO_3_-N (△), NH_3_-N (●); (**C**) TP (■), TDP (□), SRP (▲).

Environmental factors (temperature, pH, TN, TDN, NO_3_-N, NH_3_-N, TP, TDP, SRP) of samples collected monthly in bloom period are shown in Figure 1. During the sampling period, the water temperature varied from 9.7 °C to 32.7 °C, and generally peaked at the end of July or beginning of August (Figure 1A). The maximum temperature was higher in 2013 than in 2012 and 2014. The pH ranged from 7.0 to 9.2, with a minimum of 7.0 in the early phase of bloom, June in 2013, which increased corresponding with high *Microcystis* biomass. Based on datasets from two years, the variation patterns of TN and TDN in 2013 tended to decrease during blooms. TN concentrations decreased from 6.3 mg·L^−1^ in June 2013 to 1.73 mg·L^−1^ in August 2013, while another peak of 5.42 mg·L^−1^ was observed in October. In 2014, TN concentrations were low from late July to late November. The average concentration of TN (1.9 mg·L^−1^) in 2014 was lower than in 2013 (2.8 mg·L^−1^), which was calculated with values of the same four months. Nitrate concentrations decreased from 0.67 mg·L^−1^ in early June 2013 to 0.10 mg·L^−1^ in early August 2013. Interestingly, nitrate concentrations were not lower in 2014 than during the same months in 2013 and ammonium concentrations were relatively higher in 2013 than in 2014. In addition, concentrations of TP, TDP and SRP, fluctuating over sampling time, ranged from 0.05 to 0.38 mg·L^−1^, 0.027 to 0.097 mg·L^−1^ and 0.015 mg·L^−1^ to 0.067 mg·L^−1^, respectively.

### 2.2. Dynamics of Abundance and Succession of Microcystis

**Figure 2:**
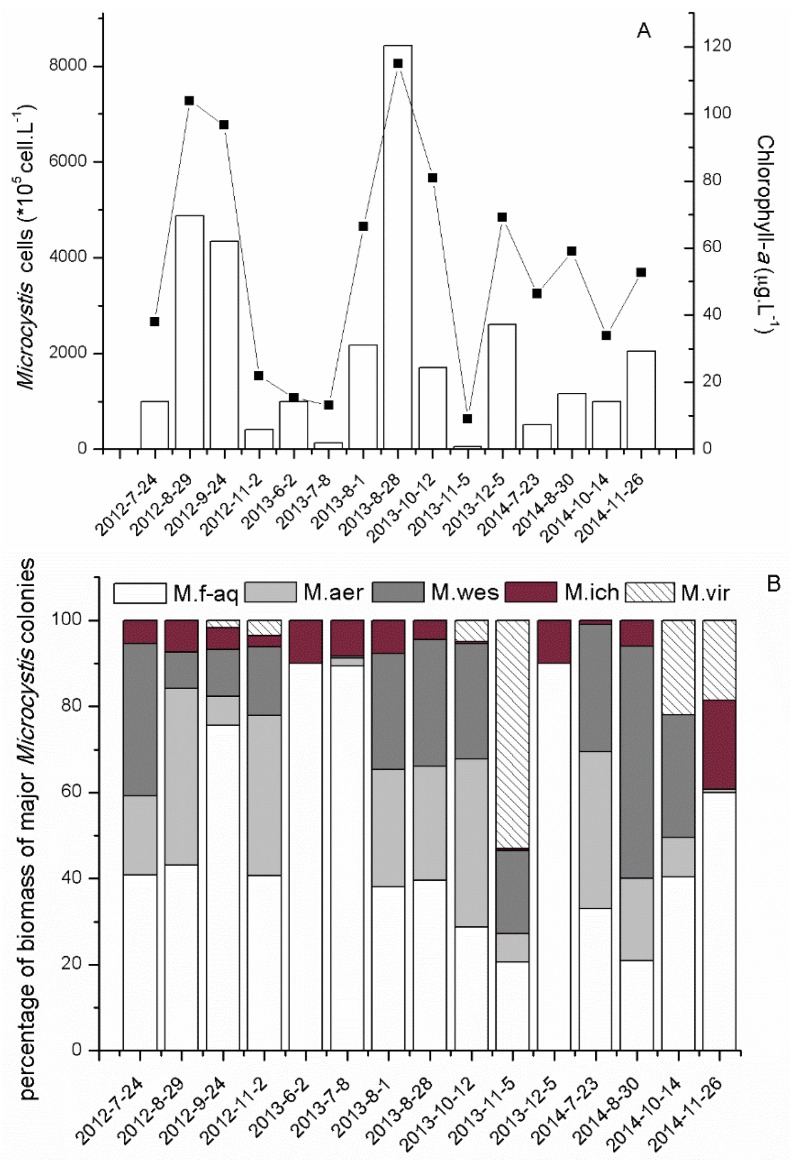
(**A**) Monthly variation of cell density of *Microcystis* (white column) and chlorophyll-*a* (■) in Meiliang Bay, N2, of Lake Taihu, from 2012 to 2014; (**B**) The percentage of biomass of the major *Microcystis* morphospecies in N2, Meiliang Bay, from 2012 to 2014.

In sampling periods, cyanobacteria populations were mainly composed of non-N_2_-fixing *Microcystis* spp., N_2_-fixing *Dolichospermum* spp. and *Aphanizomenon* spp. The abundance of *Microcystis*, the most dominant genus in the lake, ranged from 5 × 10^6^ to 8 × 10^8^ cells·L^−1^ in the three years in site N2. The Chl-*a* concentration ranged from 9.1 to 115 µg·L^−1^ (Figure 2A). The bloom of *Microcystis* comprised at least five different *Microcystis* morphotypes, *M. flos-aquae*, *M. aeruginosa*, *M. wesenbergii*, *M. viridis* and *M. ichthyoblabe*. The percentage of abundance of each *Microcystis* morphospecies during the sampling period was shown in Figure 2B. During the period of blooming, it was found that the pattern of succession of *Microcystis* morphotypes followed a trend: *M. flos-aquae* as major morphotype usually appeared at the onset of *Microcystis* bloom and sustained throughout the year. Subsequently, *M. flos-aquae*, *M. aeruginosa* and *M. wesenbergii* coexisted from August to November. Towards the decline of the bloom, *M. viridis* was either in large or low quantities in October and November.

### 2.3. Dynamics of Toxic Microcystis

The potential microcystin-producing *Microcystis* genotypes (*mcyB*) and total *Microcystis* (16S rDNA) were independently detected by qPCR in all samples. Highly significant linear curves between the initial DNA (equivalent to cell numbers) and the *C_t_* values were obtained. Regression equations were *y* = 33.127 − 3.3236*x* (*R*^2^ = 0.9977, *n* = 6) for 16S rDNA and *y* = 35.518 − 3.485*x* (*R*^2^ = 0.9999, *n* = 6) for *mcyB*, respectively, where *y* is the *C_t_* at the set fluorescence threshold level and *x* is the amount of DNA (expressed as log_10_ cell number equivalents). The amplification efficiency of both are >90% (Table 1).

**Table 1:** Primers used in this study.

Target Gene	Sequence (5′–3′)	Length (bp)	Reference	Efficiency
16S rDNA	ATGTGCCGCGAGGTGAAACCTAAT	200	Neilan *et al.*, 1997 [42]	99.9%
	TTACAATCCAAAGACCTTCCTCCC			
*mcyB*	CCTACCGAGCGCTTGGG	78	Kurmayer *et al.*, 2003 [11]	93.6%
	GAAAATCCCCTAAAGATTCCTGAGT			

**Figure 3:**
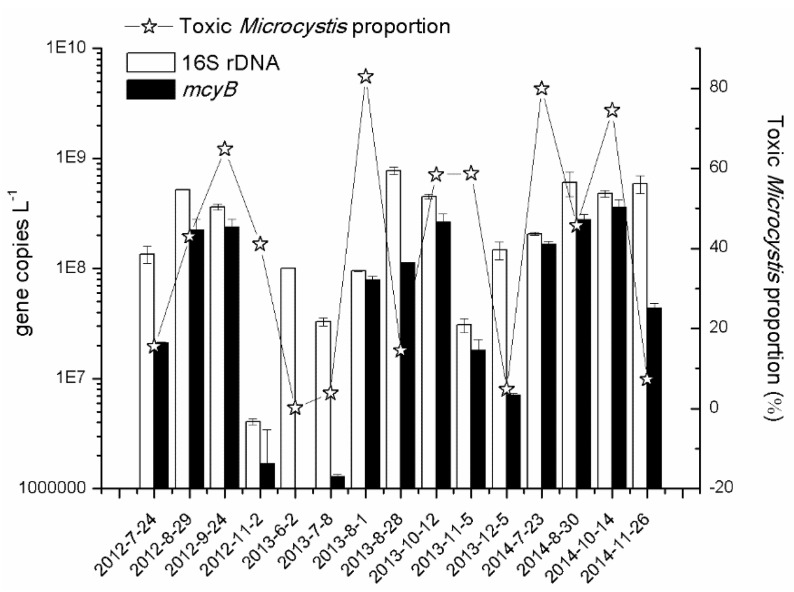
The dynamic of *Microcystis* (16S rDNA) and its toxic genotypes (*mcyB*) characterized by molecular analysis in N2, Lake Taihu in 2012, 2013 and 2014. The toxic *Microcystis* proportion was calculated using the ratio of *mcyB* to 16S rDNA.

*Microcystis* 16S rDNA copy numbers ranged from 4.1 × 10^6^ copies·L^−1^ to 7.7 × 10^8^ copies·L^−1^ (Figure 3). The copy number of *mcyB* ranged from 1.8 × 10^5^ to 3.6 × 10^8^ copies·L^−1^; the highest value of *mcyB* copy was found in September or October in all three years. Using the PCR of the *mcyB* and 16S rDNA, the relative proportion of *mcyB* genotype was estimated, ranging from 0.2% to 83% in N2. In general, the proportion of toxic *Microcystis* was relatively high from August to October in each year. At other periods, non-toxic *Microcystis* occurred in large proportions.

### 2.4. Microcystin Content of Field Samples and Cultivated Morphospecies

Dry-weight-specific MC-RR and MC-LR were detected by HPLC analysis in phytoplankton cells collected with a 40-µm plankton net. The sum of MC-RR and MC-LR were roughly considered as total microcystin. At the site of N2, total microcystin content ranged from 45 to 1664 µg·g^−1^ (Table 2). The peaks of microcystin concentrations per unit dry weight were observed in November 2012, November 2013 and October 2014, respectively. The maximum value was 1664 µg·g^−1^ in November 2013. The average microcystin (MC) concentrations were 733 µg·g^−1^, 844 μg·g^−1^, 870 μg·g^−1^ during the same period of 2012 to 2014, respectively.

On average, total microcystin was composed of 58% RR and 42% LR in N2. The percentage of RR (the average of data from July to December) was 60% in 2012. In 2013 and 2014 it was slightly less, being 53% and 54%, respectively.

**Table 2:** Microcystins content, and the percentage of RR in N2 during bloom seasons.

Site	Sampling Date (Year-Month-Day)	RR (µg/g)	LR (µg/g)	MCs (µg/g)	RR (%)
N2	2012-7-24	220.5 ± 19.3	143.7 ± 5.6	363.2 ± 74.3	61
	2012-8-29	672.8 ± 178.2	349.3 ± 45.5	1022.1 ± 146.7	66
	2012-9-24	251.6 ± 38.5	155.0 ± 111.5	406.6 ± 22.8	62
	2012-11-2	615.4 ± 23.3	525.7 ± 127.6	1141.1 ± 104.3	54
	2013-6-2	78.9 ± 68.9	14.9 ± 15.0	93.8 ± 53.9	84
	2013-7-8	250.9 ± 2.9	145.5 ± 37.1	396.4 ± 62.7	63
	2013-8-1	359.9 ± 47.3	356.4 ± 63.2	716.3 ± 110.5	50
	2013-8-28	165.1 ± 2.9	173.5 ± 5.6	338.6 ± 8.5	48
	2013-10-12	769.9 ± 4.0	686.7 ± 81.5	1455.6 ± 77.5	52
	2013-11-5	838	826	1664	50
	2013-12-5	ND	45	45	ND
	2014-7-23	676.7 ± 173.3	642.0 ± 125.2	1318.7 ± 298.5	51
	2014-8-30	193.3 ± 11.8	152.4 ± 15.7	345.7 ± 27.5	55
	2014-10-14	769.6 ± 121.1	617.1 ± 50.3	1386.8 ± 171.4	55
	2014-11-26	229.2 ± 9.6	202.3 ± 3.5	431.5 ± 13.0	53

ND: below detection limit.

### 2.5. The Variations in the Microcystis Community and their Correlation with Environmental Factors

**Figure 4:**
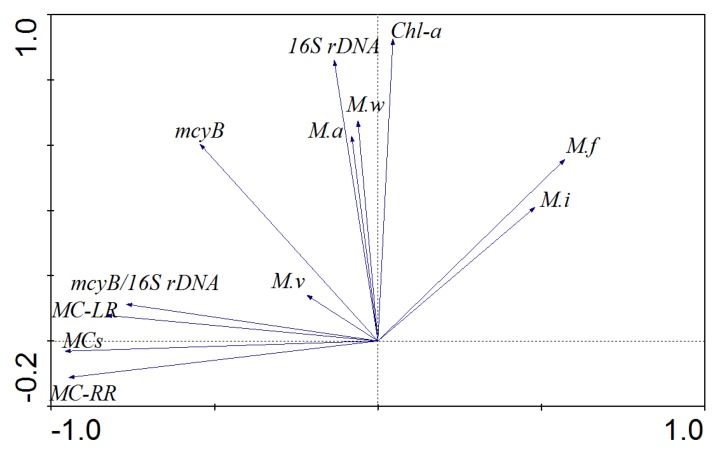
Principal component analysis plot displaying the correlation between *Microcystis* morphospecies, gene copies and MCs.

The PCA was performed using morphospecies abundances, gene copy numbers and MC concentrations as variables. The first component (PC1) and second component (PC2) combined accounted for 82.1% of the data variation (PC1 = 67.5%; PC2 = 14.6%). It was clearly demonstrated that *M. aeruginosa* and *M. wesenbergii* correlated positively with total *Microcysits* (16S rDNA), while *M. flos-aquae* and *M. ichthyoblabe* appeared to correlate negatively with microcystins (Figure 4). In addition, *M.viridis* appeared to be responsible for a small portion of the toxic potential of the samples based on PCA analysis (Figure 4) and its variation (Figure 2).

Spearman analysis was used to assess the potential effects of physical-chemical factors on total *Microcystis* and the toxicity of *Microcystis*. There was no clear relationship between nutrients concentration and total microcystin content. However, the abundance of *Microcystis* 16S rDNA showed a positive correlation with TP (*p* < 0.01) and SRP (*p* < 0.05), and *mcyB* genotype showed a significant positive correlation with TDP (*p* < 0.01) and SRP (*p* < 0.01). The results indicate that *Microcystis* (16S rDNA copies) was positively correlated with *Microcystis* cell numbers (*p* < 0.01) (Table 3).

**Table 3:** Correlation between physical-chemical factors and *Microcystis* 16S rDNA gene, *Microcystis mcyB* gene and microcystins.

Variable	*Microcystis* 16S rDNA (Gene Copies/L)	*Microcystis mcyB* (Gene Copies/L)	Microcystins (µg/g)
*Microcystis* cells (cells/L)	0.664 **	−0.248	−0.371
TN (mg/L)	0.055	0.055	−0.079
TDN (mg/L)	0.236	−0.340	0.224
NO_3_^−^ (mg/L)	−0.249	−0.173	0.012
NH_4_^+^ (mg/L)	−0.309	−0.136	0.100
TP (mg/L)	0.755 **	0.518	−0.273
TDP (mg/L)	0.589	0.767 **	0.420
SRP (mg/L)	0.715 *	0.784 **	0.374
WT (°C)	0.068	0.186	−0.018
pH	0.329	0.436	−0.118

* Correlation is significant at *p* < 0.05; ** Correlation is significant at *p* < 0.01.

## 3. Discussion

Ranked as the third largest shallow lake in China, Lake Taihu has become one of the principle focuses of the study of cyanobacterial blooms in large shallow lakes in the world. This is a consequence of the high intensity and frequency of bloom outbreaks, and massive research funding from central and local government. Over the last decade, knowledge of the dynamics of toxic *Microcystis* and toxins has been increasing, leading to a much better understanding of the lake in comparison with other lakes. Our study demonstrated that toxic *Microcystis* generally coexisted with non-toxic *Microcystis* strains in the entire *Microcystis* population. Based on data between July and December, the average percentage of toxic *Microcystis* was 41%, 43% and 52% from 2012 to 2014, respectively. In comparison, the percentage was usually <10% during the declining or early stages of bloom. It was thus conspicuous that toxic strains were more likely to dominate during the sustaining stage of bloom. Several studies in Lake Taihu have also shown that the occurrence of toxic genotypes was often associated with the period of heavy bloom [38,43,44]. Similar findings were also reported in Lake Chaohu [45] and Lake Dianchi [46]. Among the studies exploring the relationship between toxic *Microcystis* genotypes and environmental factors, Rantala *et al.* [47] reported that an increasing abundance of toxic *Microcystis* genotypes correlated with eutrophic conditions. The concentrations of phosphorus correlated strongly with the abundance of *Microcystis* and toxic *Microcystis* in Lake Erie (USA) [48], and toxic *Microcystis* cells increased with increasing levels of phosphorus and temperature [15,44,45]. In addition, Otten *et al.* [41] found that a low N: P ratio (*i.e.*, sufficient DTN and DTP) were more likely to promote toxigenic *Microcystis* strains, based on a comparison of the spatial distribution of toxic genotypes in Lake Taihu during 2009 and 2010. In our study, a relationship between toxic *Microcystis* genotype and phosphorus concentration was also found. Copy abundance of *Microcystis* 16S rDNA showed a significantly positive correlation with TP (*p* < 0.05), and the *mcyB* genotype showed a significantly positive correlation with TDP (*p* < 0.01) (Table 3). Interestingly, data from THL-5 (31.41°N, 120.19°E), a site near our sampling area, indicated Chl *a* significantly correlated with TP during 2005 to 2013 (Figure S1).

During the three years of this study, a distinctive pattern of morphospecies succession existed in the lake. Normally, *M. flos-aquae* and *M. ichthyoblabe* dominated in the early stages of bloom; subsequently, *M. aeruginosa* and *M. wesenbergii* began to increase and co-dominate in summer and autumn, whereas *M. viridis* was often found in October and November. This phenomena was partially evidenced by the study of Zhu *et al.* [49]. However, it should be stressed that *M. flos-aquae* could proliferate either in large or small quantities throughout the entire bloom period. A previous study from our group [30] illustrated that, from June to December in 2005, the dominant species changed in the following pattern: *M. flos-aquae* + *M. aeruginosa − M. wesenbergii* − *M. flos-aquae* + *M. aeruginosa*. Thus, this similarity in the pattern of succession of morphospecies from 2005 until 2014 suggested that the succession of *Microcystis* morphospecies is not only seasonally distinctive, but also annually consistent. Based on the dynamics of toxic *Microcystis* morphospecies in our study, it was observed that the dominance of *M. flos-aquae* and *M. ichthyoblabe* coincided with a quite low percentage of toxic genotypes, whereas the presence of *M. flos-aquae*, *M. wesenbergii* and *M. aeruginosa* often coexisted during bloom peak and showed no clear correlation with the percentage of toxic genotypes. There were also reports exploring the correlations between morphospecies and toxic genotypes of *Microcystis*, Kurmayer *et al.* [28] found that in Lake Wannsee in Germany, most colonies (73%) of *M. aeruginosa* contained the *mcyB* gene, whereas only 16% of the *M. ichthyoblabe* colonies and no colonies of *M. wesenbergii* resulted a PCR product of *mcyB*. The same researchers expanded their surveys across nine European countries and further showed that *M. aeruginosa* was high in potential microcystin-producing species, and that colonies of *M. ichthyoblabe* and *M. viridis* contained 20% fewer copies of the *mcyB* gene. However, other studies showed that high microcystin concentration coincided with the dominance of the toxic *M. viridis* [25,50] and that all strains of *M. viridis* are microcystin producers [51,52]. We observed that a high percentage of a toxic genotype and high microcystin content corresponded with a high prevalence of *M. viridis*. Thus, we speculated that *M. viridis* is a high-toxicity strain in Lake Taihu. To test whether *Microcystis* morphospecies could be indicative of toxicity, strains of *M. flos-aquae* and *M. aeruginosa* from Lake Taihu and *M. viridis* from Lake Dianchi were isolated and analyzed for their microcystin content. Strains of *M. flos-aquae* produced trace amounts of MC or no toxin, whereas *M. aeruginosa* and *M. viridis* produced large amounts of MC (Table S1). This approach, to some extent, supported the relationship between morphospecies and toxic genotype which had been observed in the field. Therefore, we propose that the morphotype of *Microcystis* may be a marker of bloom toxicity, as the pattern of morphospecies succession was distinctive and consistent in Lake Taihu.

The average MC content was, on dry weight basis, 733 µg·g^−1^, 844 µg·g^−1^ and 870 µg·g^−1^ (data from July to November) in N2 in 2012–2014, respectively. Correspondingly, the percentage of toxic genotype was found to be 41%, 43% and 52% from the same period, indicating that unit dry weight MC correlates with the percentage of toxic *Microcystis*. In 2005, the average MC was ~700 µg·g^−1^ (from June to December) in the nearby site [34], suggesting that on an annual basis, the toxicity of bloom in the northern lake area was relatively stable or possibly increasing over the past ten years. However, the toxicity fluctuated widely on a monthly basis. This has also been reported by other studies in Taihu Lake. For instance, the toxin concentration was ~2.0 to 9.6 µg·g^−1^ in Meiliang Bay in 2006 [18], 28.7 µg·g^−1^ in the northern area during the summers of 2009 and 2010 [41], and ~7 µg·g^−1^ (the average value was calculated from July to October) in Meiliang Bay in 2009 [38]. In general, MC concentration was closely correlated with the biomass of the toxin-producing species [14,15,17,48,53]. We also found that MCs positively correlated with *mcyB*/16S rDNA, but not with nutrients (N&P).

Our previous studies [30,34] and other studies [36,37] of Lake Taihu illustrated that MC-RR and MC-LR were the two major variants of MC, with MC-RR accounting for >50% of total MC. From 2012 to 2014, we observed a slight increase in the percentage of MC-LR, which might be associated with reduced surrounding nitrogen. The fact that the gradual decrease of TN concentration over the last decade in northern part of Taihu (Figure S2) did not coincide with a decrease and/or distinctive changes in cyanobacterial bloom biomass, toxicity or even with pattern of succession indicates the complicated relationships between nitrogen and biotic parameters. Future research in Lake Taihu should pay more attention to this issue. In summary, our findings increase the understanding of the dynamics of toxic *Microcystis* and morphospecies, and are relevant to and useful for the long-term management and mitigation of cyanobacterial blooms and to the improvement of water quality in general. Our study described the pattern of *Microcystis* morphospecies succession and the dynamics of toxic *Microcystis* over three years of investigation. As the dynamics of algal blooms can change rapidly in large shallow lakes such as Lake Taihu, sampling frequency at monthly intervals will miss some potential patterns. Therefore, future studies should utilize short high frequency sampling in order to fully understand the short and long-term dynamics of this dynamic system.

## 4. Experimental Section

### 4.1. Study Site and Sampling

**Figure 5:**
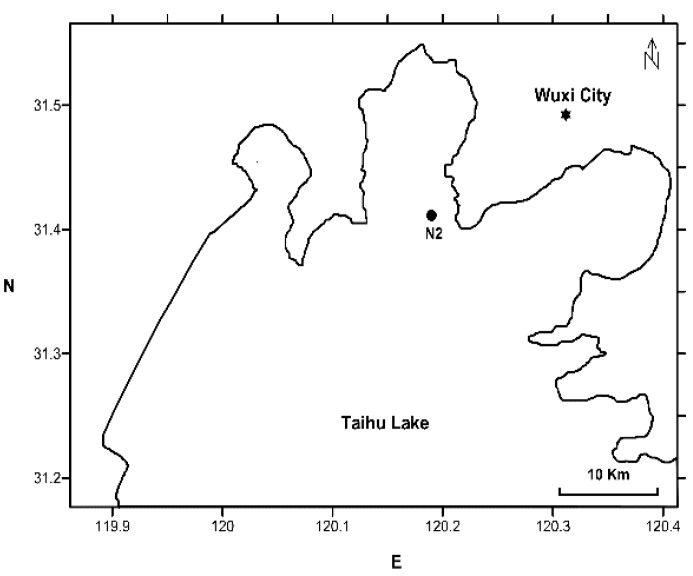
Location of the sampling site N2 (black circles) in Meiliang Bay, Lake Taihu.

The large, shallow Lake Taihu is located near Wuxi city in Jiangsu province, China. The surface area is ~2238 km^2^ and the mean depth is ~1.9 m [33]. Samples were collected four times in Meiliang Bay N2 (31.41°N, 120.19°E) (Figure 5) from July to November 2012; seven times from June to December 2013 and four times from July to December 2014 in N2, Meiliang Bay.

Water samples were collected from 0.5 m depth of surface layer. One liter of water was collected and preserved with 1% Lugol’s iodine solution for identification of the phytoplankton assemblage. Water samples for *Microcystis* DNA extraction and nitrogen-phosphorus nutrients index were transported into the lab in an ice box.

Algae samples were collected from surface water using a phytoplankton net (40-µm mesh) and were washed in freshwater to eliminate cell-bound nutrients effect. They were then stored in 50-mL sterile polyethylene bottles and frozen in solid CO_2_.

### 4.2. Physicochemical Parameters and Nutrients

Physicochemical parameters, including temperature and pH, were determined using the YSI multi-parameter water quality Sonde (6600 V2, Yellow Springs Instruments, Yellow Springs, OH, USA). The chemical variables, such as total nitrogen (TN) and dissolved total nitrogen (DTN) (GB 11894-89), total phosphorus (TP), dissolved total phosphorus (DTP) and soluble reactive phosphorus (SRP) (GB 11893-89), nitrate (NO_3_-N) (HJ/T 346-2007), nitrite (NO_2_-N) (GB 7493-87), ammonium (NH_3_-N) (HJ 535-2009), were measured according to Chinese standard methods. Water samples were filtered onto a 1.2-µm nominal pore-size polycarbonate GF/C (47-mm diameter Millipore) membrane and extracted by 90% acetone; the concentration of chlorophyll-*a* (Chl-*a*) was determined by spectrophotometry [54].

### 4.3. Identification and Counting of Microcystis Morphospecies

*Microcystis* morphospecies were classified according to morphology (Komárek and Komárková, 2002) with fresh samples collected using a plankton net (25 μm diameter). Samples of each *Microcystis* morphotype colonies were picked out from fresh sampling, fixed using 0.1 mL Lugol′s iodine solution and shaken until the colonies turned into single-cells. These were counted using a microscope (Eclipse E200, Nikon, Tokyo, Japan). The average cell numbers of each colony type were used to calculated the relative biomass of each type of *Microcystis* colony, and the abundance of each major *Microcystis* morphospecies was calculated using the average cell numbers of each morphospecies [30].

### 4.4. DNA Extraction and qPCR

Water samples were filtered onto a 0.22-µm nominal pore-size polycarbonate membrane (XingYa factory, Shanghai, China) until high phytoplankton biomass had accumulated on the membrane, and genomic DNA was extracted by a water DNA kit D5525-01 (Omega Bio-tek, Norcross, GA, USA) according to its protocol.

To quantify the abundance of toxic *Microcystis* spp. and total *Microcystis* spp. in Lake Taihu, a standard curve preparation real-time PCR assay was performed using 16S rRNA and *mcyB* gene primers. A 20-mL culture solution of *M. aeruginosa* PCC7806, containing 2.7 × 10^7^ cells·mL^−1^, was filtered through a 0.22-µm nominal pore-size polycarbonate membrane (XingYa factory, Shanghai). Extracted DNA from the membrane was prepared using five dilution gradients, ranging from 10 to 10^5^ dilutions of template DNA (equivalent to 5.4 × 10^7^ cells to 5.4 × 10^3^ cells). The standard curve was based on DNA concentrations (equal to cell numbers) with the threshold cycle (*C_t_*) of the diluted samples.

A quantitative real-time PCR assay was performed in a volume of 20 µL, with 10 µL SYBR-Green (Toyobo, Osaka, Japan), 8 µL sterile water, 0.5 µL each of forward and reverse primer and 1 µL template DNA (~100 ng): *mcyB* and 16S rDNA primers see Table 1. Amplification was performed in a Bio-Rad real-time Thermos Cycler using the following protocol: for *mcyB*, initial denaturation at 95 °C for 1 min, followed by 40 cycles of 95 °C for 15 s, 59 °C for 30 s (decreasing by 0.5 °C each cycle) and 72 °C for 30 s. For 16S: preheating for 1 min at 95 °C, followed by 40 cycles of 25 s at 95 °C, 30 s at 53 °C and 60 s at 70 °C. All samples were amplified in triplicate. After PCR, fluorescent melting curve analysis was performed by gradually increasing the temperature from 65 °C to 95 °C at a rate of 0.1 °C·s^−1^.

### 4.5. Extraction and Determination of Particulate Microcystins

Freeze-dried algal cell samples (about 50 mg) were magnetically stirred with 5% acetic acid for 30 min, centrifuged at 7000 rpm for 10 min and the supernatant transferred to a bottle; the algal residues were extracted with 90% (*v*/*v*) aqueous methanol for 30 min. Methanol extract was concentrated to 1–2 mL by rotary concentration and all extract were through C18 Sep-pak cartridges. Elution was performed on a C18 Sep-pak column with 10–15 mL methanol and evaporation to dry. Finally, microcystin was eluted in solutions with 1 mL 50% (*v*/*v*) chromatographic pure methanol (Thermo Fisher Scientific, Waltham, MA, USA) and stored at −20 °C for HPLC analysis. The microcystin concentration was determined by HPLC [55].

### 4.6. Statistical Analysis

The principal component analysis of the *Microcystis* morphospecies, gene copies and MCs, was performed using CANOCO 4.5 (Microcomputer Power, Ithaca, NY, USA). Spearman’s correlation analysis was used to determine the relationship between microcystin content, *Microcystis,* toxic *Microcystis* and physical-chemical factors using SPSS statistics software version 20 (SPSS Inc., Chicago, IL, USA). A correlation was considered significant at *p* < 0.05.

## 5. Conclusions

Based on three consecutive years of investigation of the dynamics of toxic genotypes, MC production and morphospecies succession, combined with the comparison of data obtained over a decade, we were able to describe the status of toxic *Microcystis* and its toxicity in the northern parts of Lake Taihu.The average proportions of toxic genotypes ranged from 41% to 52% during the bloom periods. The pattern of morphospecies succession was seasonally distinct and annually consistent. The MC concentration of dominant morphospecies was relatively stable. Therefore, we propose that morphospecies could indicate toxicity of a bloom, especially during the early or late stages. The toxicity of the bloom was not only dependent on the abundance of toxic *Microcystis*, but also related to MC production and the composition of MC congeners. Based on historical and current data, we can conclude that the annual bloom toxicity of Lake Taihu was relatively stable or has possibly increased during the last decade.

## Supplementary Materials

The following are available online at www.mdpi.com/2072-6651/8/1/23/s1.
