# Supplementary material for: Multi-Year Assessment of Toxic Genotypes and Microcystin Concentration in Northern Lake Taihu, China
Source: Toxins (Basel). 2016 Jan 15;8(1):23. doi: 10.3390/toxins8010023 (PMC4728545; doi:10.3390/toxins8010023)
Supplement: Supplementary file 1 [file toxins-08-00023-s001.pdf]

Supplementary Materials: Multi-Year Assessment of Toxic Genotypes and Microcystin Concentration in Northern Lake Taihu, China

Lili Hu, Kun Shan, Lizhou Lin, Wei Shen, Licheng Huang, Nanqin Gan and Lirong Song

Table S1. Dry weight microcystin of isolates of *Microcystis* in culture.

Strain	RR (μg/g)	LR (μg/g)	MCs (μg/g)
M.flos-aquae TH-1	26.0	ND	26.0
M.flos-aquae TH-2	ND	4.5	4.5
M.flos-aqua TH-3	ND	ND	ND
M.aeruginosa TH-1	2573.8	891.5	3465.3
M.viridis DC-1	436.0	930.9	1366.9
M.viridis DC-2	360.1	533.8	893.9

ND: below detection limit.

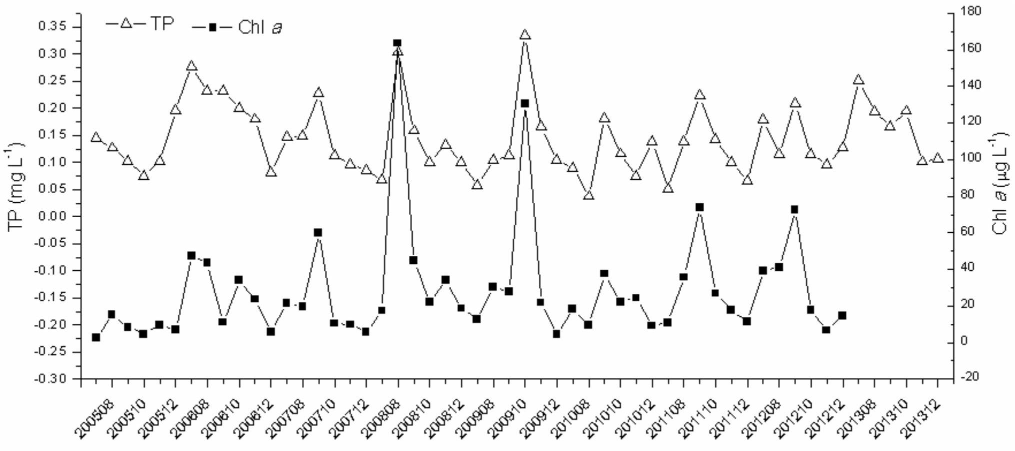

Figure S1. Inter-annual variation of TP and Chl-*a* between July and December in THL5, Meiliang Bay, Lake Taihu.

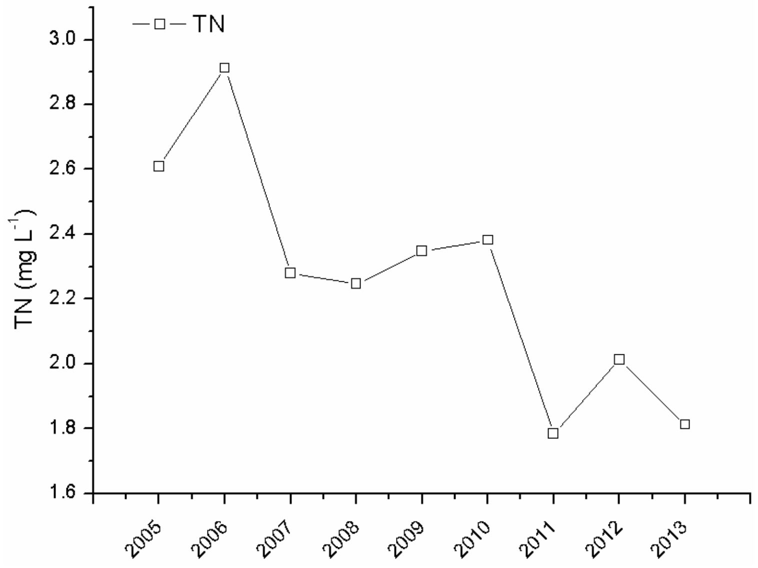

Figure S2. Annual mean values of TN in THL5, Meiliang Bay, based on data from summer to late autumn (July–December).
